# Cultural Threads: An Afrocentric Paradigm for Integrating Social Justice Principles in the Practice of Family Therapy in Africa

**DOI:** 10.1111/jmft.70091

**Published:** 2025-11-14

**Authors:** Ronald Asiimwe, Doneila L. McIntosh, Rehema Gathumbi Nyambura, Rosco Kasujja

**Affiliations:** ^1^ Department of Family Social Science University of Minnesota, Twin Cities Minneapolis Minnesota USA; ^2^ Department of Human Development and Family Science University of Georgia Athens Georgia USA; ^3^ Private Practice Nairobi Kenya; ^4^ Department of Mental Health Makerere University Kampala Uganda

## Abstract

Family therapy has been slowly but steadily growing on the African continent. Considering Africa's rich yet complex economic, political, and sociocultural history, it is essential for family therapy practitioners to integrate social justice (SJ) principles into their research, training, and practice of family therapy. By doing so, we can help foster more inclusive, culturally responsive, and meaningful support for the historically underrepresented communities of African descent. More importantly, we recommend that addressing SJ issues in the practice of family therapy in Africa be rooted in Afrocentric frameworks of care and practice. Drawing on existing literature, we introduce the African‐Centered Wellness Model as a foundational framework for advancing socially just family therapy with African families. To demonstrate how the framework translates into clinical practice, two authors present case vignettes from their clinical work. A discussion of implications for integrating the principles of the model into research, training, and practice follows.

The practice of systemic family therapy is rooted in the understanding that mental, emotional, and psychological well‐being cannot be separated from the relational, sociocultural, and political contexts in which individuals and families exist. Recent family therapy literature has underscored the critical importance of integrating social justice (SJ) principles into practice, arguing that overlapping identities (e.g., religion, social class, gender, sexual orientation, and others) can create varying experiences of marginalization and privilege (AAMFT 2023; Cutts 2013; Morrison et al. 2022). Within the family therapy field, it is broadly understood that taking an SJ perspective is essential for ensuring that available care and services meet and address the needs of clients, particularly those from culturally diverse backgrounds (Almeida and Tubbs 2020).

In Africa, where historical legacies and contemporary structures continue to shape family dynamics and mental health experiences, a socially just approach to family therapy is not only beneficial but also essential (Lombard and Twikirize 2014; Pillay 2020). Our main argument in this article is that engaging in ongoing discourse regarding SJ issues is vital for the growing profession of systemic family therapy across the continent. Without this lens, existing family therapy models risk ignoring and maybe misrepresenting the critical and nuanced sociopolitical and cultural forces that impact African families both on the continent and in the diaspora. Furthermore, unlike previous literature from Africa, which has addressed SJ largely within the fields of social work, school psychology, and clinical psychology (Ahmed and Pillay 2004; Pillay 2019), this article brings a much‐needed focus to family therapy, a field that is growing at a faster rate across Africa (Asiimwe et al. 2021).

A crucial component of this article is our proposed African‐Centered Wellness Model (ACWM) (Garrett‐Akinsanya 1998), which serves as the foundation for shaping our argument as well guiding the discussion set forth in the subsequent pages of this article. By proposing an Afrocentric framework rooted in resonant Afrocentric cultural values, spiritual traditions, and communal resilience, we aim to address the longstanding absence of African indigenous perspectives in mainstream family therapy practice (Nwoye 2015). We believe that failure to meaningfully integrate African‐centered paradigms into family therapy risks perpetuating cultural disconnection and clinical irrelevance. Furthermore, we integrate with our own clinical experiences and existing literature, to highlight how contemporary mental and relational health issues of most African individuals and families are shaped by SJ issues related to but not limited to, colonial legacies, war and organized violence, economic disparities, and gender‐based inequalities, and others. Thus, the purposes of this article are twofold: (1) to identify and discuss key SJ issues affecting families in Africa using existing literature and (2) to offer and discuss the utility of an actionable, Afrocentric model in therapeutic practice with families in both mainland Africa and African families in the diaspora.

To our knowledge, this is the first article to critically examine SJ issues in African family therapy practice through an Afrocentric lens. We seek to raise awareness and equip family therapists, and other mental health practitioners, with the tools to enact transformative, contextually relevant, and socially just systemic care with African families and communities, in the now and in the future. We also seek to bridge the gap in practice between family therapy with African clients on the African continent and those in the diaspora, such as those living in the United States and other developed countries.

As authors, we approach the discussion on SJ issues in Africa with an acknowledgment of the continent's immense cultural and linguistic diversity (54 countries, over 1000 ethnic groups) while also recognizing shared commonalities in values, beliefs, and practices across^1^ African countries, particularly those in the sub‐Saharan region. Therefore, we caution against imposing external viewpoints or universalizing assumptions that may not align with African contexts, as the perspectives we share in this paper are informed by existing literature and anecdotal data from our own clinical work. The article sets forth by first acknowledging the historical undermining of local knowledge systems during colonialism and the continued challenge of integrating indigenous ways of knowing in contemporary family therapy practices. By centering African perspectives in family therapy discourse, we aim to challenge colonial legacies and promote culturally relevant and contextually appropriate mental health practices. Throughout the process of writing this article, we reflected on whether SJ issues in African societies mirror those in Western societies or have distinct historical and cultural roots shaped by the unique contexts of each region. We also wondered whether these issues were human rights concerns or shaped by a Western lens, which we acknowledge we have been exposed to, to larger extent. These curiosities coupled with our unique cultural intersectional lived and clinical experiences as family therapy scholars of African descent guided our exploration of the current literature that shaped this study.

Our hope is that the article will encourage readers to critically reflect on the evolution of family therapy in Africa, by considering both the historical and contemporary challenges that shape its practice now and in the future. We invite practitioners from all cultural backgrounds to engage in thoughtful discussions about the SJ issues facing contemporary diverse families, the role of historical events like colonialism and racism, and how these systems of injustices currently shape and/or will continue to shape the future of family therapy practice in the multicultural diverse settings such as those in Africa.

## 1 Conceptualizing SJ in Africa: A Systems Theory Perspective

SJ is such a multifaceted concept, often difficult to define comprehensively, yet most scholars agree on five core principles of SJ: *access* (equal availability of resources), *equity* (fair opportunities despite historical injustices), *diversity* (inclusive representation in positions of power), *participation* (community involvement in decision‐making), and *human rights* (protection of individual freedoms and dignity) (Cutts 2013). From a systems theory perspective, these principles reflect how individual, familial, and community challenges are deeply interconnected with and are influenced by larger societal structures (Gómez‐Carrillo and Kirmayer 2023).

Within the African context, mental health and relational issues cannot be separated from historical and systemic influences. For example, the history of colonialism, socioeconomic disparities, wars and organized violence, and the imposition of Western ideologies and religion have shaped current mental health problems across many African communities (Asiimwe et al. 2021). These systems of inequality, rooted in colonial and postcolonial histories, continue to influence the present‐day struggles, such as access to health care, education, and economic opportunities of many African families and communities both on the continent and in the diaspora. The psychological distress arising from these injustices manifests in family systems, where mental health issues, such as trauma, grief, and dysfunction, often reflect larger societal struggles.

In line with Winter and Hanley (2015), SJ in therapy should therefore not remain an abstract notion but be actively translated into practice. In this context, the framework we present, the ACWM, provides a culturally responsive approach for doing so, specifically by emphasizing the importance of cultural resonance in health and wellness. By centering African worldviews, traditions, and values, the model acknowledges the vital role these cultural elements play in shaping individual and collective mental and relational health. Thus, it is essential to situate this discussion within a broader systemic context to understand how deeply interwoven these issues are across the personal, familial, and societal levels.

## 2 Contextualizing and Exploring African Diversity

Africa is one of the most culturally diverse continents on the planet, with over 1.4 billion people, represented by many ethnic groups and languages (World Bank 2024). This immense diversity shapes Africa's social, economic, and political systems, which in turn influence cultural traditions, values, and interpersonal relationships across its 54 countries. Broadly, Africa can be conceptualized in four large blocks which include, *Anglophone* Africa (referring to countries like Uganda, Kenya, South Africa, Nigeria, Ghana, Zimbabwe, Zambia, and others that were colonized by Britain), *Francophone Africa* (referring to countries colonized by France and Belgium, such as Ivory Coast, Rwanda, Burkina Faso, Togo, Senegal, and others), *Portuguese Africa* (e.g., Mozambique, Angola, and Madagascar), and Arab Africa (e.g., Algeria, Egypt, Tunisia, Morocco, Libya, Sudan, etc.). Other blocks of Africa include countries such as Eritrea, Somalia, and Ethiopia (that were not directly colonized by foreign powers) and Liberia, which was part of the American Colonization Society Project (Scruggs 2010).

Even within each African country, there is significant immense cultural, linguistic, and ethnic diversity. In Uganda, for example, the Baganda speak Luganda and maintain a centralized monarchy through the Buganda Kingdom, while the Basoga and Acholi speak different languages and follow distinct cultural traditions. Similarly, Kenya, with a population exceeding 50 million, has over 40 ethnic groups. The Kikuyu, part of the Bantu group, are predominantly farmers, while the Maasai, from the Nilotic group, are pastoralists. Kenya has made strides toward SJ through the 2010 Constitution, which laid the foundation for promoting human rights and equality (The Constitution of Kenya 2010). Despite this progress, challenges such as poverty, gender‐based violence, youth unemployment, and corruption persist. Institutions like the Kenya National Commission on Human Rights (Kenya National Commission on Human Rights 2021) and civil society groups are instrumental in advocating for marginalized communities. Nigeria is another example of African countries exemplifying deep ethnic diversity. For example, the Hausa people of northern Nigeria follow Islamic customs, the Yoruba in the southwest blend Christianity with traditional practices, while the Igbo in the southeast maintain unique cultural beliefs rooted in African spirituality. Green (2013) attributes Africa's diversity to historical influences like geography, colonialism, the slave trade, and urbanization, which reinforced ethnic divisions across the continent.

In the postcolonial era, issues such as urbanization and migration have redefined Africa's ethnic diversity. Despite colonial influences, many Africans have preserved their languages and traditions. Present‐day Africans are multilingual and tend to speak their native languages alongside colonial languages, like, English, Portuguese, or French (Asiimwe et al. 2021). Today, Africa remains a mosaic of cultures, even within single nations, while at the same time maintaining the *Ubuntu* (a shared sense of humanity, harmony, and interconnectedness; Nussbaum 2003). This analysis highlights the resilience and adaptability of African identities in preserving their rich heritage amid globalization and modernization. The examples presented demonstrate that even within a single African nation, the diversity of languages, traditions, and cultural practices can be profound. As observed above, cultural disparities impact SJ issues since historically, traditional norms have tended to create inequities in political participation, health care, and education. To promote diversity and equity, we must find a balance between protecting cultural heritage while at the same time furthering socioeconomic development and human rights.

Building on this understanding of Africa's cultural diversity and resilience, it is essential to explore how African‐centered paradigms can offer valuable frameworks for addressing SJ issues in contemporary contexts, particularly the growing field of family therapy on the continent. By integrating African philosophies into discussions of SJ, we can better understand how these paradigms can contribute to fostering more inclusive and culturally responsive approaches to addressing challenges faced by African communities globally.

## 3 The Rationale for Integrating Afrocentric Perspectives in Conceptualizing SJ as It Relates to Couple and Family Therapy in Africa

Historically, Western scientific ideals which have dominated psychotherapy practice in most of Africa have been criticized for being elitist, Eurocentric, and disconnected from local realities (Leopeng 2019; Vorhölter 2024). Some scholars have argued that for family therapy to gain legitimacy with African people, it must integrate the cultural, spiritual, and sociopolitical narratives of African families and communities (Bakker and Snyders 1999). Today, Africa faces numerous SJ challenges, to mention a few such as poor health systems, gender inequality, and discrimination against sexual and gender minorities (SGMs), while also simultaneously possessing vast economic potential. With a population of approximately 1.4 billion and a median age of 19, the continent's youth are driving economic growth and transformation (UNDP 2020; World Bank 2021).

Indeed, Africa's immense cultural diversity and the increasing participation of African practitioners in the training, research, and practice of family therapy underscore the increased need for an Afrocentric perspective on SJ. Contemporary African family therapy scholars like Augustine Nwoye (2018) have highlighted the potential for family therapy to foster social change and resilience among Africans by emphasizing indigenous healing practices and integrating African worldviews into therapeutic models to address psychosocial challenges in ways that are both contextually relevant and empowering. This would enable practitioners to empower families toward change while challenging systemic injustices, at the same time. Failure to do so risks silencing African social values (e.g., communism and relational harmony) and lived experiences, thereby continuing to render indigenous knowledge invisible (Almeida et al. 2017). Moreover, it perpetuates colonial‐era assumptions of Western superiority and limits the growth of indigenous frameworks, which can lead to poorly prepared, socially unconscious therapists and ineffective practices (Zimmerman and Haddock 2013). Furthermore, without culturally resonant frameworks, clinical practice may alienate African communities and hinder cross‐cultural research and collaboration. To address these issues, we now introduce an ACWM as a framework through which we can advance the SJ agenda within family therapy in Africa.

## 4 The African‐Centered Wellness Model

**Figure 1:**
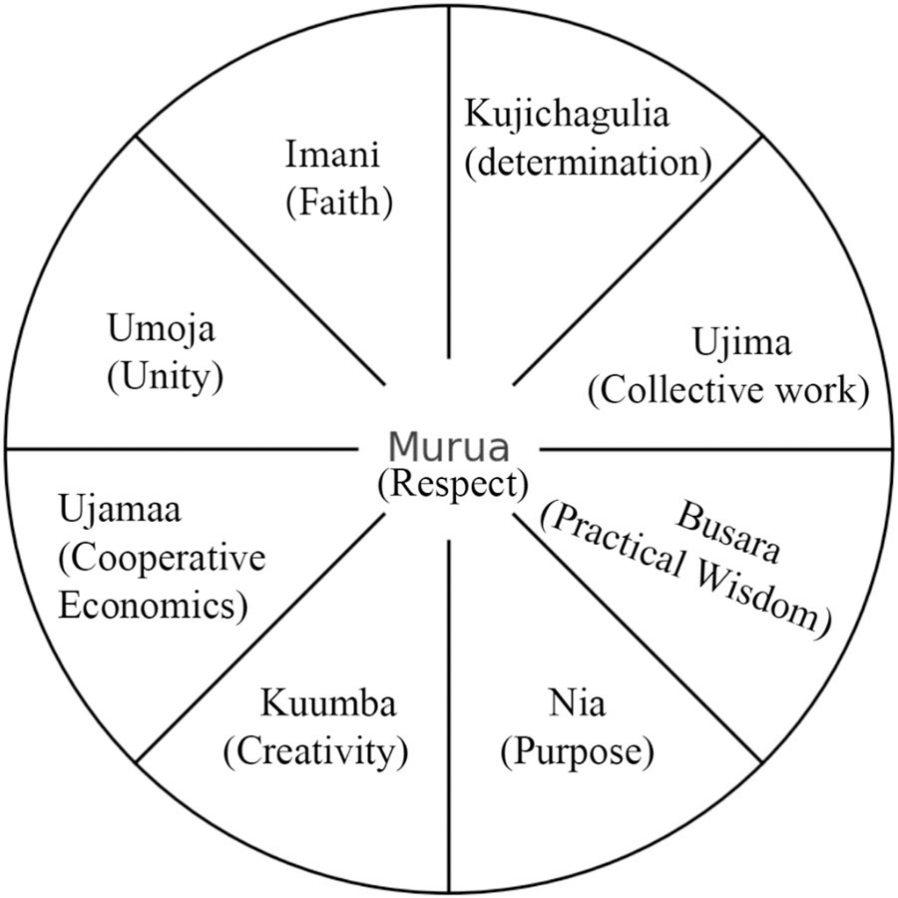
The African‐centered wellness model (Garrett‐Akinsanya 1998).

The ACWM is a culturally grounded, systemic framework designed for working with individuals of African descent. Developed by Garrett‐Akinsanya (1998), this framework emphasizes the holistic integration of body, mind, and spirit within a communal and ecological context. Additionally, the model recognizes the influence of cultural heritage, ethnic identity, and spirituality on well‐being (Grills et al. 2018; Myers 1991; Nobles 1992). Rooted in Afrocentric psychology, the model's main aim is to promote psychotherapy practices that affirm and integrate the clients' traditions, beliefs, and values (Grills et al. 2018). Although this model has been widely applied across African communities on the continent and in those in the diaspora, its potential for addressing SJ issues in family therapy remains underexplored in published literature. Given Africa's complex multicultural landscape, which is shaped by historical as well as ongoing, sociocultural, and political influences, the model offers a culturally resonant framework for family therapists to address mental and relational health challenges through the lens of cultural wisdom and contextual understanding. This model is established on the Nguzo Saba (seven principles of ancient African wisdom) (Karenga 1988), and outlines eight interconnected dimensions of wellness, using Swahili terminology to integrate cultural values and enhance relevance for Africans globally (Figure 1).

Typically identified as a wheel, with *Murua* (respect) as its central spoke, the model emphasizes mutual respect for oneself and others in the community as the foundation for holistic well‐being. *Nia* (purpose) represents a vocational calling that brings joy and fulfills both divine purpose and collective empowerment, which in turn supports vocational and occupational wellness. *Umoja* (unity) emphasizes striving for and maintaining unity within the family, community, and beyond to foster mental, relational, and emotional wellness. *Kuumba* (creativity) celebrates the beautification of African cultures through music, art, storytelling, theater, writing, and other forms of cultural expression, while also valuing play and experiential learning. *Ujimaa* (cooperative economics) is a dimension focusing on building and maintaining financial resources that support the family and community at large. *Imani* (faith) highlights the practice of spirituality or religion, which tends to be practiced through communal rituals, ancestral veneration, respect for elders, nature reverence, oral traditions, music, dance, drumming, sacred ceremonies, divination, and the honoring of spirits. *Busara* (practical wisdom) values the integration of indigenous and academic to contribute to intellectual wellness. *Kujichagulia* (self‐determination) promotes the collective definition, naming, and advocacy for oneself and one's community, particularly in resisting the remnants colonization. Finally, *Ujima* (collective work and responsibility) underscores the importance of building and maintaining community while accepting shared responsibility for addressing challenges within relationships, families, and communities, which supports social wellness.

## 5 Bridging Theory and Practice: African‐Centered Family Healing Across Three Cases

To further demonstrate how our proposed ACWM can be implemented in practice, we present clinical vignettes drawn from the authors' work with African clients across both continental and diasporic contexts. These cases build on SJ issues to demonstrate how family therapists can translate the model's core principles into therapeutic action. Across many African families and communities, tensions frequently arise from the intersection of traditional norms and evolving contemporary realities, such as shifting gender roles, attitudes toward sexual orientation, migration‐related stressors, and generational differences in worldviews. These dynamics often create psychological and relational strain for African individuals and families navigating identity, belonging, and power dynamics in complex social environments. It is worth noting that while most of our focus in this article is on family therapy in continental Africa, we acknowledge that two of the cases we chose involve Africans living in the United States, where broader societal attitudes, particularly regarding topics like sexual orientation and educational choices, may generally be more open and accepting than in certain African countries. These differing cultural climates could likely shape client experiences, decisions, and therapeutic outcomes. We, therefore, caution readers to approach these cases with an appreciation for their distinct sociocultural contexts, while also recognizing that cultural commonalities may persist across African families despite geographic differences (e.g., one case in Uganda and two in the United States). Recognizing these contextual differences is essential to understanding how societal norms can influence both the therapeutic process and the strategies clients use to navigate identity and life choices.

The three client cases featured include a Liberian American family living in a midwestern US state, a Nigerian male professional dealing with minority stress and historical trauma in the United States, and a Ugandan family negotiating gendered expectations. Of the 100s of clients we have served as family therapists, the two of us (the first author R.A. and second author D.L.M.) selected these three cases to highlight the versatility, flexibility, and depth of our proposed model in addressing such complex client challenges across diverse contexts, in a socially just manner. Each case is grounded in therapeutic practices that affirm the core tenets of the proposed ACWM. In the first two case studies, the ACWM was introduced to clients during the intake session, as therapist/second author D.L.M., uses the model to assess clients during the intake. Clients received an explanation of the model, how it is utilized and incorporated in identifying therapeutic goals and treatment. In the third case, the model was integrated into the sessions as it became apparent to therapist/first author R.A. that the model suited the needs of the clients.

To maintain client confidentiality, we have used pseudonyms and altered some minor details to conceal client identity. It is important to note that these vignettes are not intended to prescribe a singular approach to SJ‐oriented therapy with African clients, nor do they imply that every session must overtly address all facets of the ACWM. Rather, they exemplify the nuanced, context‐sensitive applications of its core principles, and most importantly, emphasize the importance of cultural humility, relational attunement, and therapeutic flexibility. When approached with respect for cultural context and attentiveness to locally relevant SJ issues, these cases illustrate therapists serving families can practice with integrity and cultural responsiveness, regardless of their own backgrounds, while promoting healing within diverse families in therapy.

### 5.1 Case 1: Rebuilding Connection Through Shared Purpose and Faith

The Sessay family, were Liberian immigrants raising their two teenage sons, Kofi (17) and Samuel (14), in the United States Deeply rooted in Christian faith, the family upholds values of education, discipline, and respect for tradition. However, they encountered increasing conflict over cultural identity and career expectations. Mr. Sessay, an engineer, placed immense value on what he referred to as “respectable” professions and discipline, while sons Kofi and Samuel envisioned paths in advocacy and creative technology, goals their father saw as risky or even inappropriate. Meanwhile, Mrs. Sessay, recovering from trauma after a workplace accident, noticed the family drifting apart emotionally. Recognizing the potential of therapy to improve her family dynamics, she initiated family therapy sessions with her sons. Mr. Sessay, on the other hand, declined to attend, initially expressing skepticism and cultural discomfort around therapy. Initial sessions with this family revealed Kofi's distress about being pressured to follow a career path that did not reflect his interests—he felt called to work in education and advocacy. Samuel, the youngest, was passionate about video game design, a dream his father often dismissed as “childish” or “too American.” Mr. Sessay frequently critiqued Samuel for “acting too Black,” using language that reflected internalized bias toward African American cultural expression.

Grounded in the ACWM, therapy began by fostering *Umoja* (unity), rebuilding unity between the mother and sons through affirming their care for one another, while respecting generational differences. Through *Kujichagulia* (self‐determination), Kofi and Samuel found the language and courage to assert their aspirations without guilt. The therapist (D.L.M.) framed the children's pursuit of self‐definition not as rebellion, but as an extension of the very resilience their parents modeled as immigrants in the United States. When Mr. Sessay finally joined therapy sessions, the turning point came through centering *Imani* (faith) and *Busara* (wisdom). The therapist reframed the father's initial hesitancy to come to therapy as normal and protective as a survivor of the Liberia civil war who immigrated to a foreign land to gain secure stability for his family. Through spiritual and cultural reflection, Mr. Sessay was invited to see how his own journey embodied *Kujichagulia* and *Ujamaa* (collective responsibility), supported by family members who believed in him.

This empathetic reframing helped Mr. Sessay to soften. He began to conceptualize his sons' dreams not as threats, but as expressions of their shared legacy of resilience, hard work, and support for one another. Family prayer returned, and respect deepened across generational lines, which all accelerated Mrs. Sessay's healing as she witnessed the family's emotional reconnection. The therapist used the family's Christian faith to deepen this message, pointing out that the Bible affirms that each person is *“fearfully and wonderfully made”* (Psalm 139:14), and that God plants a unique purpose in every individual. Therapy illuminated a path where purpose (*Nia*), faith, and self‐expression could coexist. This process helped to bridge the parents' deeply held African traditional values while honoring and supporting the needs of their American‐born sons.

### 5.2 Case 2: Reimagining Identity Through Murua and Kuumba

Tunde, is a 35‐year‐old successful Nigerian software engineer in the United States. He is the eldest of three sons. Tunde presented therapy with increasing burnout, anxiety, and unresolved trauma linked to his suppressed sexual identity and family tensions. He believes that his challenges were deeply intertwined with longstanding tensions in his family, given his sexual orientation, as an African Gay man, which he has not openly disclosed to all his family but believes is a source of silent judgment and emotional distance. As the eldest son in a family with rigid expectations around masculinity, achievement, and heteronormativity, Tunde feels unseen and invalidated, especially in comparison to his brother, a physician who embodied the family's ideals.

Therapy began with *Kuumba* (creativity), reframed as a liberatory process of envisioning life beyond shame. The therapist D.L.M. used guided imagery and narrative work, to help explore what freedom felt like if his decisions were rooted in self‐love. This was not merely about coming out, but about reconnecting with a fuller self. To counter years of internalized judgment, the therapist grounded her alliance in *Murua* (sacred respect). Therapy was redefined not as a Western act of “fixing” Tunde, but as a deeply African practice of restoration and self‐honor. Murua allowed Tunde to release self‐condemnation and enter a space of affirmation. He began to understand that respecting himself did not mean rejecting his family but rather reclaiming the right to exist wholly and authentically. Throughout therapy sessions with Tunde, *Ujima* (collective responsibility) became the ethos of the sessions. He learned to view therapy as a collaborative journey, not a solitary burden. He allowed himself to be witnessed, supported, and guided through moments of emotional regression and healing. Over time, his self‐narrative shifted from one of deficiency to one of emerging wholeness. The cultural and therapeutic frameworks intertwined to support a process where Tunde could integrate his various identities, African, queer, spiritual, intellectual, into a coherent self.

### 5.3 Case 3: Negotiating Daughters' Education Through Cultural Legacy

The last case we present is one of a Ugandan family who worked with the first author (R.A.) a few years ago. The Kizito family was a low‐income family in Uganda comprising a father, 50‐year‐old Mr. Kizito, 40‐year‐old Mrs. Kizito, and two adolescent daughters, Sammie (18 years old) and Tasha (15 years). They sought therapy citing ongoing conflicts regarding the girls' educational trajectory. They reported that there were ongoing tensions around the father's traditional values, which demanded that girls prioritize mastering domestic responsibilities and preparing for early marriage, while the mother quietly supported the girls' education, but felt powerless to advocate openly for fear of disrupting household harmony. Using the ACWM, I began by grounding our sessions in Umoja (unity) to affirm the family's shared value of cohesion, while validating the conflict as a sign of care, not division. Drawing on Nia (purpose), I facilitated a conversation with the father around legacy and purpose, exploring how his daughters' education could serve the collective good. Through Kujichagulia (self‐determination) and Imani (faith), we invoked stories of strong women leaders in Ugandan history (such as former vice president, Specioza Kazibwe, 1994–2003 and former speaker of the Ugandan parliament between 2011 and 2021). These were influential female figures that Mr. Kizito was familiar with as a Ugandan and could see her girls emulate them. Over the course of our work, the father began to reframe his fears as protective love, which eventually led to Busara (practical wisdom) where the parents began to work collaboratively to plan for the girls' schooling responsibilities while striving to preserve certain empowering cultural values to support their daughters' wholistic growth.

## 6 Connecting the Dots: A Summary of Implications for Clinical Practice

Having introduced three practical case studies that employed the ACWM, we now examine key SJ issues (through the lens of the proposed model) that necessitate ongoing consideration for the developing field of systemic family therapy across the continent. In the three cases presented, therapy became more than just symptom relief; it became a sacred, socially just, culturally responsive, and restorative journey drawing on core principles of the ACWM. These principles not only grounded therapeutic work in African cultural values but also created pathways to healing that resonated across generations and continents. Whether in urban America or Uganda, these illustrations affirm that African cultural wisdom is not separate from mental health but rather, it is fundamental to it.

This article arrives at a crucial time when discussions of SJ are beginning to take central stage in African family therapy literature and discourse. Yet, the same systemic issues that dominate global discourses, the likes of gender inequality, access disparities, trauma, racial/ethnic discrimination, are also deeply relevant across African communities. Thus, the ACWM offers a coherent, culturally grounded framework for tackling these challenges, to foster culturally attuned and socially just care amidst structural inequalities.

### 6.1 Access to Trauma‐Informed, Culturally Relevant Care

Arguably, the most pressing SJ issue in African contexts is access to quality mental and relational health care. Systematic reviews by Essien and Asamoah (2020) and Aguwa et al. (2023) highlight several barriers to care, including stigma, inadequate infrastructure, and a shortage of trained professionals. Particularly in many rural areas of Africa, individuals often rely on established traditional structures of care, which often include, consultations with respected elders, traditional healers and local herbalists, religious figures, and others, before seeking the help of a mental health professional. While these structures of care have been relied upon for many years, they tend to delay care and can at times, compound the client's symptoms. Even among wealthier families, religious leaders are often the first line of consultation in cases of relational distress, which underscores the cultural salience of religion in Africa. We thus argue that family therapists (and truthfully all mental health professionals) across the continent, and those working with African populations globally, must advocate for policies that ensure culturally relevant, affordable, and accessible services. Drawing on Nwoye's (2018) concept of therapists as agents of social change, the ACWM provides a framework for therapists to support efforts toward equity in mental health care. Clinicians are encouraged to move beyond treating isolated symptoms and instead consider broader systemic influences, such as historical trauma, structural inequality, and cultural dislocation. Therapists must also engage in advocacy work to help shape mental health policies that bridge traditional healing and modern therapeutic care, such as family therapy, which is still a new kid on the block in many African settings (Asiimwe et al. 2021). This approach will ensure that no individual or family is left behind or untreated.

Furthermore, given the widespread impact of conflict, war, and colonial legacies in Africa, trauma‐informed care must also be culturally grounded. Armed conflict, political instability, and displacement have had devastating psychosocial impacts across the continent. Many families face unprocessed trauma, with limited access to trauma‐informed and relationally oriented care. While trauma‐focused interventions like Narrative Exposure Therapy and Eye Movement Desensitization and Reprocessing have been effective in some contexts (Koebach et al. 2021), they largely focus on treating individual symptoms, neglecting relational and communal healing. The ACWM emphasizes relational well‐being and the interconnectedness of individuals, families, ancestors, and spiritual systems. We encourage therapists to consider how trauma reverberates through family systems, not just individuals, and to include traditional beliefs and communal practices in their interventions. Culturally attuned trauma care emphasizes collective healing, spiritual wellness, and relational restoration can more effectively support families in postconflict or high‐adversity settings. As such, there is a need for equipping mental health providers in African settings with skills to offer relational trauma therapy that reflects the communal healing frameworks central to African contexts.

### 6.2 Therapist Training and Culturally Responsive Practice

As cultural adaptation becomes a global priority in mental health practice, therapist training must be reoriented to include culturally responsive frameworks (Knudson‐Martin et al. 2019; Seponski et al. 2013). Given the African cultural diversity discussed earlier, the growing practice of family therapy among African communities must reflect the diverse cultural beliefs, family systems, and community values of the continent. This is especially critical given that historically, and even today, many current mental health interventions in Africa are based on predominantly individualistic Western models of psychotherapy, which tends to neglect African relational norms or community structures (Bakker and Snyders 1999; Asiimwe et al. 2021). Importantly, models emerging from historically underrepresented contexts, such as the proposed ACWM, can play a vital role in training culturally competent clinicians, especially those from Africa or those intending to work with African clients. This approach is useful for preparing therapists to deliver interventions that reflect the lived realities and cultural strengths of African families while at the same time addressing contemporary SJ challenges.

### 6.3 Family Focused Interventions for Youth Mental Health

With much of the African population under 18 (United Nations 2022), youth mental health must be prioritized. Jörns‐Presentati et al. (2021) conducted a systematic review of 37 studies, in which they reported high prevalence rates of depression (26.9%), anxiety (29.8%), behavioral problems (40.8%), posttraumatic stress disorder (PTSD) (21.5%), and suicidal ideation (20.8%) among sub‐Saharan adolescents. These rates surpass those found in many high‐income regions, yet mental health infrastructure in most of Africa remains severely underdeveloped. The literature shows that untreated mental health problems among youth lead to academic challenges, substance abuse problems, and social withdrawal, which impact not only the individual but also the family unit as well (Jörns‐Presentati et al. 2021). Given the appalling statistics above, addressing the mental health challenges of African youth requires culturally adapted family therapy interventions that integrate African ideas and values. Thus, we encourage therapists to find ways to integrate principles from the proposed model to foster safe, culturally affirming environments for youth at risk of various mental health challenges. By leveraging African principles like respect for elders, communal connections, and interdependence, therapists can help families build protective relational networks that support the mental, emotional, and relational well‐being of the young people of Africa.

### 6.4 Preventing Child Marriage Through Systemic Engagement

Child marriage is another issue with deep relational and developmental implications for families and communities in Africa. Research by Yaya et al. (2019) reported that child marriage affected 54% of girls across 34 sub‐Saharan African countries, with rates as high as 81.7% in countries like Niger. Early marriage is associated with increased fertility, terminated pregnancies, early childbirth, and diminished opportunities for economic advancement. It also undermines girls' educational and economic potential as well as exposes girls to power imbalances in intimate relationships and intimate partner violence. Family therapists working through the African‐Centered Wellness lens can engage families and communities to shift and gently challenge (like in the third case with the Ugandan family) norms and beliefs and prevent early marriages. Rather than only highlighting the harm, such as lost educational opportunities, relational distress, and low self‐esteem, therapists can frame prevention within cultural narratives of faith, protection, care, and collective responsibility. Through collaborating with parents and elders, practitioners can promote wellness and empowerment without dismissing local values. This approach has the potential to foster mutual learning, healing, and transformation for families and communities.

### 6.5 Cultural Wisdom for Navigating Issues of Gender Inequality

It is our belief that gender inequality and power imbalances in African societies require a culturally sensitive, non‐polarizing approach. In most of Africa, gender disparities manifest in unequal access to education, employment, and health care, while economic instability exacerbates relational stress, domestic violence, and child neglect (Karoui and Feki 2018). While we acknowledge that certain patriarchal norms can be harmful, they also function within cultural systems that provide structure and meaning for many families and communities. Rather than adopting an adversarial stance, we encourage therapists to apply cultural humility and practical wisdom, key principles in the ACWM, to open dialog and foster inclusivity. This “Both/And” perspective respects multiple truths and could avoid the potential harmful consequences of unintentional alienating one gender over the other. By positioning family therapists as facilitators of safe, reflective conversations, this approach could help families transform without fracturing.

### 6.6 Addressing the Needs of SGMs

Discrimination against SGMs remains a persistent violation of human rights across many African countries. For example, homosexuality is criminalized in 33 out of 55 African countries (Mugisha 2024), and this social stigma continues to marginalize LGBTQ+ individuals, further exacerbating mental and relational health challenges among individuals that belong to these communities. Studies show that SGMs face high rates of psychological distress, PTSD, and depression due to systemic discrimination and violence (Müller and Daskilewicz 2018; Pulerwitz et al. 2024). In Kenya, one study found that over half of 527 surveyed SGMs reported PTSD symptoms, and 26.1% experienced depression (Harper et al. 2021). These alarming statistics highlight that working with SGMs in Africa demands a balance of courage, cultural knowledge, and clinical ethics. Given the legal and societal discrimination SGMs face in many African countries, open advocacy may not always be feasible, and in worst cases could lead one to harsh legal consequences. However, (CFTs) couple and family therapys can create affirming therapeutic spaces that offer validation, safety, and support for SGM clients by intentionally applying the principles of the ACWM, especially the Kujichagulia (self‐determination) dimension, which emphasizes naming, defining, and advocating for one's community, while at the same time, actively resisting oppression. For example, therapists could gently challenge harmful societal narratives against SGM clients within the therapeutic process, promote community dialog, and affirm ideas of shared humanity (i.e., Ubuntu) and Umoja (Unity). The therapist's role as a culturally grounded bridge‐builder becomes essential in transforming stigma into empathy, even in highly conservative and discriminatory contexts.

### 6.7 Collaborative Advocacy and Policy Engagement

Ultimately, we believe that systemic issues like poverty, child marriage, and gender inequality require more than therapy, they demand structural change. Family therapists are well placed to advocate for equitable policies, work alongside community leaders, and contribute to public education efforts. Rooted in systemic thinking, our training equips us to engage complex social systems and cocreate sustainable solutions with communities. Thus, therapists should leverage their position to promote empowerment, gender equity, and child protection while upholding cultural values that foster cohesion and healing.

## 7 Training and Research Implications

As family therapy gains traction in Africa, CFT education and training in Africa must evolve and be rooted in the continent's cultural and sociopolitical realities. While the emergence of CFT graduate programs, especially in Kenya, signals progress, these programs must intentionally prepare therapists to address systemic inequalities through SJ‐oriented curricula. We agree with Zimmerman and Haddock (2013), that therapists must be equipped to challenge ingrained biases shaped by colonialism, patriarchy, and homophobia. Thus, programs must emphasize cultural competence, antioppressive frameworks, and Afrocentric paradigms such as the proposed ACWM to challenge the status quo. Curriculum redesign should prioritize African epistemologies, experiential learning in diverse settings, and critical engagement with global literature. As Wampler and Patterson (2020) note, systemic therapy provides tools for contextual thinking, but without culturally and contextually attuned training, therapists risk replicating oppressive structures. Relational and systemic approaches, when culturally grounded, are ideally suited to support African families navigating sociopolitical and historical adversity. Integrating Afrocentric paradigms, such as the ACWM, could further strengthen training, bridge generational divides, and foster resilience.

Research is equally vital in all these endeavors. As Pillay (2020) notes, advancing SJ requires building a culturally relevant evidence base. The ACWM offers a foundation for exploring culturally grounded, strength‐based interventions. However, no empirical evidence currently supports the model's usefulness in examining the mental and relational health and well‐being of African families and communities. To advance the science, African CFT researchers must investigate the model's practical and empirical applications, develop locally grounded approaches, and document traditional practices already addressing family mental health.

In sum, the future of family therapy in Africa depends on integrating Afrocentric training and research frameworks that are socially transformative, culturally relevant, and empirically supported to empower therapists to meet the unique needs of African families.

## 8 Conclusion

The proposed ACWM provides not only a theoretical anchor but also a practical guide for family therapists seeking to engage in socially just family therapy practice with clients from the diverse African contexts. The implications we highlight in this paper show that culturally grounded therapy does not have to be at odds with modern practice. Rather, we see it as a Both/And, in that, when traditional values are integrated with intentionality, creativity, and innovativeness, therapists can help families reclaim their heritage, foster resilience, and navigate today's mental and relational health challenges with dignity and strength. The task for African CFTs, but really for all CFT serving clients from culturally diverse backgrounds globally, then, is not merely to adapt Euro‐American centric models but to lead with frameworks that affirm the values of diverse clients, promote systemic healing, and build equitable futures from the inside out. By embracing this mindset, therapists serving culturally diverse African families in Africa and globally are poised to see a transformative shift in their practice.

## Endnotes

1 In this article, the prefix “African” is used to indicate generalizable theoretical abstract theme (s) that are dominant and enduring across Africa. By no means, it does not imply that Africa is monolithic but a recognition of the ever‐changing nature of cultural traditions. It is used in a similar manner; the prefix “Western” is often used in literature to refer to certain dominant themes in Western cultures and thought. It does not imply that all cultures or even traditions in the “West” are the same, but rather that some aspects of the traditions are shared among Western societies.
